# Performance Survey and Comparison Between Rapid Sterility Testing Method and Pharmacopoeia Sterility Test

**DOI:** 10.1007/s12247-017-9303-z

**Published:** 2017-12-05

**Authors:** Adriana Bugno, Deborah Pita Sanches Saes, Adriana Aparecida Buzzo Almodovar, Kamal Dua, Rajendra Awasthi, Daniela Dal Molim Ghisleni, Marici Tiomi Hirota, Wesley Anderson de Oliveira, Terezinha de Jesus Andreoli Pinto

**Affiliations:** 1 Adolfo Lutz Institute, 355 Dr. Arnaldo Avenue, São Paulo, 01246-000 Brazil; 2 Discipline of Pharmacy, Graduate School of Health, University of Technology Sydney, Sydney, NSW 2007 Australia; 3 School of Pharmacy and Biomedical Sciences, The University of Newcastle, Newcastle, Callaghan, NSW 2308 Australia; 4 School of Pharmaceutical Sciences, Shoolini University, Solan, Himachal Pradesh 173229 India; 5 NKBR College of Pharmacy & Research Centre, Meerut, Uttar Pradesh 245206 India; 6 São Paulo University, Avenida Professor Lineu Prestes, 580, São Paulo, 05508-000 Brazil

**Keywords:** Rapid microbiological method, Culture media, Sterility test, Sterility testing performance

## Abstract

The sterility test described in pharmacopoeial compendia requires a 14-day incubation period to obtain a valid analytical result. Therefore, the use of alternative methods to evaluate the sterility of pharmaceuticals, such as the BacT/Alert® 3D system, is particularly interesting, because it allows a reduced incubation period and lower associated costs. Considering that the BacT/Alert® 3D system offers several culture media formulations developed for this microbial detection system, the present study was aimed to evaluate and compare the performance of BacT/Alert® 3D with the pharmacopoeial sterility test. There was no significant difference between the ability of the culture media to allow detection of microbial contamination. However, the rapid sterility testing method allowed a more rapid detection of the challenge microorganisms, which indicates that the system is a viable alternative for assessing the sterility of injectable products.

## Introduction

The sterility test is considered one of the oldest and most well-established microbiological tests used in the pharmaceutical field. It ensures the quality and safety of products and has been used with minor modifications since its introduction in the British Pharmacopoeia in 1932. The compendia sterility test is a presence-absence test in which the turbidity of the culture media is indicative of microbial growth and verified by visual inspection. The culture media used in the sterility test are fluid thioglycollate medium (FTM) and soybean-casein digest medium (SCDM). FTM is used to detect aerobic and anaerobic microorganisms, and SCDM is used to detect aerobic bacteria and fungi. The incubation period of both media is 14 days, and each medium has a specific incubation temperature [1–3]. Considering the long incubation period required by the pharmacopoeial method to obtain an analytical result, the interest of the pharmaceutical industries in evaluating and validating rapid technologies suitable for sterility testing and for the isolation and detection of microorganisms has increased [1–11]. The BacT/Alert® 3D system is one of these rapid technologies that may be useful for the sterility testing of pharmaceuticals. In this system, microbial detection is based on the colorimetric detection of CO_2_ produced as a result of microbial growth in liquid culture media. The BacT/Alert® 3D system is automated and is capable of incubating, shaking, and continuously monitoring culture media, with readings taken every 10 min throughout the entire incubation period. In 2004, the BacT/Alert® 3D system received FDA approval for use in assessing the sterility of short half-life products used in cell therapy, namely Carticel [6, 9, 12].

The pharmacopoeial sterility test requires the use of two culture media, FTM and SCDM. The BacT/Alert® 3D system offers formulations to improve the detection of aerobic and anaerobic microorganisms. BacT/Alert® SA and BacT/Alert® FA media were introduced to the clinical field for the detection of aerobic microorganisms, whereas BacT/Alert® SN and BacT/Alert® FN media were introduced for the detection of anaerobic microorganisms. In the field of processed or aseptically prepared food products, the culture media BacT/Alert® *i*AST, BacT/Alert® *i*NST, and BacT/Alert® *i*LYM were introduced for the detection of aerobic and anaerobic microorganisms and fungi, respectively. However, there is not BacT/Alert® media development specifically for the microbial detection in pharmaceutical products.

The use of rapid microbiological methods for sterility testing relies on their ability to recover and detect microorganisms occasionally present in pharmaceuticals and on microbial viability by multiplication in liquid culture media with performance equivalent to the compendial methods [3, 4, 9–11]. Pharmacopoeial compendia [3] and alternative microbiological method validation guide [11] indicate which validation parameters should be evaluated according to the type of microbiological test; for qualitative tests such as the sterility test various parameters like specificity and detection limit must be assessed.

The specificity of the alternative method is based on its ability to detect the different microorganisms potentially present in the product. Thus, the growth-promoting ability of the culture media should be evaluated against strains of microorganisms suitable for use in pharmacopoeial growth promotion test [1–3, 5, 10, 11]. The detection limit refers to the smallest number of microorganisms that can be detected under the stated experimental conditions [3, 11].

Considering the possibility of using the BacT/Alert® system in pharmaceutical sterility testing, the aim of the present study was to evaluate and compare the microbial detection efficiency of the BacT/Alert® system for the detection of aerobic and anaerobic microorganisms with that of the pharmacopoeial sterility testing.

## Materials and Methods

### Culture Media

The BacT/Alert® culture media were evaluated by comparison with the pharmacopoeial media (FTM and SCDM). All the culture media were provided by BioMerieux (France).

The BacT/Alert® 3D culture bottle provide both a culture media with nutritional and environmental conditions for microorganisms and a microbial detection system, which utilizes a liquid emulsion sensor (LED) at the bottom of each culture bottle visibly change color when the pH changes due to the rise in CO_2_ as it is produced by microorganisms.

The BacT/Alert® standard media (BacT/Alert® SA and BacT/Alert® SN) were introduced for the detection of aerobic and anaerobic microorganisms, respectively and are formulated by supplemented tryptic soy broth (TSB), whereas BacT/Alert® FAN media (BacT/Alert® FA and BacT/Alert® FN) are formulated by peptone-enriched TSB supplemented with brain heart infusion (BHI) solids and activated charcoal, which neutralize antimicrobials present in the sample.

The BacT/Alert® media for processed or aseptically prepared food products, BacT/Alert® *i*AST and BacT/Alert® *i*NST, are formulated by supplemented TSB—as the BacT/Alert® standard media—whereas BacT/Alert® *i*LYM, introduced for the detection of fungi specifically, are formulated by supplemented carbohydrate medium.

Although the media BacT/Alert® SN, BacT/Alert® FN, and BacT/Alert® *i*NST present same formulation that the media BacT/Alert® SA, BacT/Alert® FA, and BacT/Alert® *i*AST, the bottles contain an atmosphere of nitrogen under vacuum that allows the detection of anaerobic microorganism.

### Microorganisms

The following microorganism strains were used as indicated in the pharmacopoeial compendia for evaluating the growth-promoting ability of the culture media: *Staphylococcus aureus* (ATCC 6538), *Bacillus subtilis* (ATCC 6633), *Pseudomonas aeruginosa* (ATCC 9027), *Clostridium sporogenes* (ATCC 19404), *Candida albicans* (ATCC 10231), and *Aspergillus brasiliensis* (ATCC 16404). All microorganisms were obtained in the form of a BioBall® SingleShot (BioMerieux, France) containing approximately 30 colony-forming units (CFUs)/unit.

The performance for microbial detection of the BacT/Alert® SA, BacT/Alert® FA, BacT/Alert® *i*AST, BacT/Alert® *i*Lym, and the conventional medium SCDM was evaluated using strains of *S. aureus* (ATCC 6538), *P. aeruginosa* (ATCC 9027), *B. subtilis* (ATCC 6633), *A. brasiliensis* (ATCC 16404), and *C. albicans* (ATCC 10231).

The performance for microbial detection of the BacT/Alert® SA, BacT/Alert® FA, BacT/Alert® *i*AST, BacT/Alert® *i*Lym, and the conventional medium FTM was evaluated using strains of *S. aureus* (ATCC 6538), *P. aeruginosa* (ATCC 9027), and *B. subtilis* (ATCC 6633).

The performance for microbial detection of the BacT/Alert® SN, BacT/Alert® FN, BacT/Alert® *i*NST, and the conventional medium FTM was evaluated using strains of *S. aureus* (ATCC 6538), *P. aeruginosa* (ATCC 9027), *B. subtilis* (ATCC 6633), *A. brasiliensis* (ATCC 16404), and *C. sporogenes* (ATCC 19404).

### BioBall® SingleShot Control

Before use in the evaluation of the media performance, eight units of each BioBall® SingleShot were tested to confirm the microbial load; four units were used on each of two different days. Each BioBall® SingleShot unit was dissolved in 1 mL of 0.9% sterile saline solution, provided by BioMerieux (France) and inoculated on: tryptic soy agar (TSA) for *S. aureus*, *B. subtilis*, and *P. aeruginosa*, with subsequent incubation at 32.5 ± 2.5 °C for 72 h; TSA for *C. albicans*, incubated at 22.5 ± 2.5 °C for 5 days; Dichloran Rose Bengal Chloramphenicol (DRBC) agar for *A. brasiliensis*, incubated at 22.5 ± 2.5 °C for 5 days; or blood agar for *C. sporogenes*, incubated at 32.5 ± 2.5 °C for 72 h under anaerobic conditions.

Plate counts were performed after incubation periods, all plates were inspected for purity, and Gram stain was performed to confirm the absence of contamination.

All this procedures was performed for all different lots used in this study.

### Preparation of Microbial Suspensions

For each challenge microorganism, four BioBall® SingleShots were dissolved in 24 mL of 0.9% sterile saline solution (BioMerieux, France). From this suspension, 8, 4, 3, 2, and 1 mL aliquots were transferred to sterile flasks, and the final volume of each flask was adjusted to 100 mL with 0.9% sterile saline solution (BioMerieux, France) to obtain suspensions with concentrations approximately of 2.00, 1.00, 0.75, 0.50, and 0.25 CFU/5 mL, respectively.

Additionally, 3 mL aliquot of the initial suspension was inoculated on TSA for *S. aureus*, *B. subtilis*, and *P. aeruginosa*, with subsequent incubation at 32.5 ± 2.5 °C for 72 h; TSA for *C. albicans*, incubated at 22.5 ± 2.5 °C for 5 days; DRBC agar for *A. brasiliensis*, incubated at 22.5 ± 2.5 °C for 5 days; or blood agar for *C. sporogenes*, incubated at 32.5 ± 2.5 °C for 72 h under anaerobic conditions, in order to verify the microbial load. Plate counts were performed after incubation periods, all plates were inspected for purity, and Gram stain was performed to confirm the absence of contamination.

### Evaluation of the Culture Media Performance

Each BacT/Alert® culture medium was compared with the pharmacopoeial media as to their specificity and ability to recover low-inoculum concentrations of the microorganism strains described in Table 1.

**Table 1:** Frequency of detection of microbial growth for each culture medium evaluated

Microorganism	Conventional media		BacT/Alert® media						
	FTM (*n* = 20)	SCDM (*n* = 20)	FA (*n* = 20)	SA (*n* = 20)	*i*AST (*n* = 20)	*i*LYM (*n* = 20)	FN (*n* = 20)	SN (*n* = 20)	*i*NST (*n* = 20)
*Staphylococcus aureus*	17 (85.0%)	20 (100.0%)	18 (90.0%)	18 (90.0%)	17 (85.0%)	00 (0.0%)	16 (80.0%)	14 (70.0%)	16 (80.0%)
*Pseudomonas aeruginosa*	12 (60.0%)	17 (85.0%)	16 (80.0%)	16 (80.0%)	17 (85.0%)	00 (0.0%)	12 (60.0%)	12 (60.0%)	12 (60.0%)
*Bacillus subtilis*	17 (85.0%)	18 (90.0%)	19 (95.0%)	16 (80.0%)	18 (90.0%)	00 (0.0%)	18 (90.0%)	17 (85.0%)	18 (90.0%)
*Clostridium sporogenes*	18 (90.0%)	NA	NA	NA	NA	NA	17 (85.0%)	16 (80.0%)	17 (85.0%)
*Candida albicans*	NA	18 (90.0%)	18 (90.0%)	18 (90.0%)	17 (85.0%)	18 (90.0%)	NA	NA	NA
*Aspergillus brasiliensis*	NA	16 (80.0%)	17 (85.0%)	17 (85.0%)	18 (90.0%)	15 (75.0%)	NA	NA	NA
*Χ* ^2^ (*p* value)			0.0215 0.8834	0.3344 0.5631	0.0852 0.7703	52.28^(1)^ < 0.05 0.0918^(2)^ 0.7901	0.0382 0.8451	0.5735 0.4489	0.0382 0.8451

*NA* not available

^(1)^Considering all microorganisms

^(2)^Considering only yeasts and molds

Aliquots of 5 mL of each microbial concentration were inoculated into two flasks of each of the culture media to be evaluated with the aid of a sterile calibrated syringe and 27-gauge needle. Aliquots of 5 mL of 0.9% sterile saline solution were inoculated into two flasks of each of the culture media to ensure the sterility of both the culture media and the 0.9% sterile saline solution. The conventional media were incubated under the conditions recommended for sterility tests: FTM at 32.5 ± 2.5 °C and SCDM at 22.5 ± 2.5 °C, and both media were evaluated daily for 5 days to verify the presence of microbial growth. The BacT/Alert® media were incubated only at 32.5 ± 2.5 °C for 5 days, and automatic readings were taken at every 10 min.

During the incubation period, all media exhibiting a positive result (turbity or positive result on BacT/Alert®) underwent Gram staining and subculture for microorganism identification.

The tests were repeated five times, on different days, until totaling 10 replicates for each type of culture medium for each microbial concentration of each challenge microorganism.

### Sterility Test

The BacT/Alert® 3D system was evaluated by comparing it to the pharmacopoeial membrane filtration sterility testing method, regarding the detection of the microorganisms used to intentionally contaminate commercial 0.9% sodium chloride solution and a commercial metronidazole solution, both in 100-mL bag.

Units of each product (P1 and P2) were artificially contaminated with a suspension containing 2 CFU/5 mL, and the same number of units were also contaminated with a suspension containing 0.25 CFU/5 mL of each microorganism strain used to evaluate the BacT/Alert® culture media.

Each of the samples was filtered onto a cellulose nitrate filter having a nominal pore size not greater than 0.45 μm and a diameter of approximately 50 mm, under aseptic conditions. After filtration of the samples, the membrane was washed three times by filtering 100 mL of Fluid A through it; then, the membrane was cut aseptically into two equal parts, and one half was transferred to conventional culture media—FTM and SCDM—that were incubated under the conditions recommended for sterility tests: FTM at 32.5 ± 2.5 °C and SCDM at 22.5 ± 2.5 °C, during 14 days. Both types of culture media were evaluated daily to detect the presence of microbial growth.

After 18 h incubation, 10-mL aliquots of the SCDM were transferred to a bottle of BacT/Alert® SA and 10-mL aliquots of the FTM were transferred to a bottle of BacT/Alert® SN; the BacT/Alert® media were incubated only at 32.5 ± 2.5 °C for 14 days, and automatic readings were taken at every 10 min.

The same procedure was performed to others pairs of BacT/Alert® media: after an 18-h incubation, 10-mL aliquots of the SCDM were transferred to a bottle of BacT/Alert® FA and 10-mL aliquots of the FTM were transferred to a bottle of BacT/Alert® FN; and 10-mL aliquots of the SCDM were transferred to a bottle of BacT/Alert® *i*AST and 10-mL aliquots of the FTM were transferred to a bottle of BacT/Alert® *i*NST. All the BacT/Alert® media were incubated only at 32.5 ± 2.5 °C for 14 days, and automatic readings were taken at every 10 min.

Negative controls consisting of filtrations of each of the matrices that had not been intentionally contaminated were included in all of the assays. Negative controls of the culture media were also used to confirm the sterility of these reagents. Positive controls of the inoculum were used to confirm their viability and ability to grow in the culture media.

All of the flasks containing culture media, both for the conventional and the BacT/Alert® 3D methods, that had microbial growth were subjected to Gram staining and subcultured to identify the microorganism.

The tests were repeated five times, on different days, until totaling 10 replicates for each product in each microbial concentration of each challenge microorganism.

### Statistical Analysis

The chi-squared test was used to compare the number of positive cultures detected in each BacT/Alert® or conventional medium (SCDM and FTM). Analysis of variance (ANOVA) Two way was used to examine the differences in the time required to detect microbial growth by each BacT/Alert® and conventional medium. The *p* value of <0.05 was considered statistically significant.

The chi-square test was used to evaluate whether the BacT/Alert® 3D system was equivalent to the conventional method. Two-way ANOVA was used to examine the differences in the time required to detect microbial growth. The *p* value of < 0.05 was considered statistically significant.

## Results and Discussion

### Specificity Evaluation of Culture Media

Specificity refers to the ability to detect a microorganism strain that may be present in a sample [3, 9, 11]. Because the sterility test is a qualitative assay, specificity was determined as the ability of the culture media to promote growth of the challenge microorganisms in suspensions with 2 CFU/5 mL and 1 CFU/mL loads (Table 1).

We did not find significant difference (*p* > 0.05) in the ability to promote the growth of aerobic and anaerobic bacteria between the BacT/Alert® FN, BacT/Alert® SN, and BacT/Alert® *i*NST media and the conventional medium FTM nor between the BacT/Alert® FA, BacT/Alert® SA, BacT/Alert® *i*AST, and TSB for aerobic microorganisms.

The BacT/Alert® *i*LYM medium showed significantly lower performance (*p* < 0.05) than the TSB medium for the detection of aerobic microorganisms. However, the ability of the BacT/Alert® *i*LYM medium to promote the growth of *A. brasiliensis* and *C. albicans* was equivalent to the TSB medium.

The search for molds and yeasts that contaminate sterile products began in 1942, when USP XII introduced a culture medium containing honey, which was replaced in 1950 by Sabouraud broth. In 1970, USP XVIII incorporated SCDM for the sterility test because of its specificity to detect fungi and its ability to allow the growth of molds, yeasts, and aerobic bacteria, replacing Sabouraud broth [13]. The BacT/Alert® *i*LYM medium, like Sabouraud broth, shows selectivity for the detection of fungi. Therefore, its use for sterility testing of pharmaceuticals is not appropriate, as it does not detect a great variety of microorganisms that may be present in a sample.

### Limit of Detection

The limit of detection refers to the smallest number of microorganisms in a sample that can be detected, but not necessarily quantitated, under the stated experimental conditions [3, 9, 11]. The detection frequency of the challenge microorganisms in suspensions with bacterial loads of 2.00, 1.00, 0.75, 0.50, and 0.25 CFU/5 mL were tested in this study, as well as the lowest concentration at which 50% positive results were obtained (Tables 2 and 3).

**Table 2:** Detection frequency of aerobic microorganism growth at five different contamination levels for each culture medium evaluated

Microorganism	Culture medium	*N* per contamination level	Contamination level (CFU/5 mL)					LOD observed (CFU/5 mL)
			2.00	1.00	0.75	0.50	0.25	
*Staphylococcus aureus*	SCDM	10	10	10	7	*6*	4	0.50
	BacT/Alert® FA	10	10	8	7	*6*	3	0.50
	BacT/Alert® AS	10	10	8	6	*5*	2	0.50
	BacT/Alert® *i*AST	10	10	7	7	*5*	3	0.50
	BacT/Alert® *i*LYM	10	0	0	0	0	0	–
*Pseudomonas aeruginosa*	SCDM	10	9	8	*6*	4	1	0.75
	BacT/Alert® FA	10	9	7	*6*	3	0	0.75
	BacT/Alert® AS	10	9	7	*7*	4	2	0.75
	BacT/Alert® *i*AST	10	10	7	*6*	3	1	0.75
	BacT/Alert® *i*LYM	10	0	0	0	0	0	–
*Bacillus subtilis*	SCDM	10	10	8	7	*6*	4	0.50
	BacT/Alert® FA	10	10	9	*7*	4	2	0.75
	BacT/Alert® AS	10	10	6	*6*	4	2	0.75
	BacT/Alert® *i*AST	10	10	8	*6*	4	2	0.75
	BacT/Alert® *i*LYM	10	0	0	0	0	0	–
*Candida albicans*	SCDM	10	10	8	*6*	4	1	0.75
	BacT/Alert® FA	10	10	8	*5*	3	1	0.75
	BacT/Alert® AS	10	10	8	*6*	3	2	0.75
	BacT/Alert® *i*AST	10	10	7	6	*6*	3	0.50
	BacT/Alert® *i*LYM	10	10	8	6	*5*	1	0.50
*Aspergillus brasiliensis*	SCDM	10	9	7	6	*5*	1	0.50
	BacT/Alert® FA	10	9	8	*6*	4	1	0.75
	BacT/Alert® AS	10	10	7	*6*	4	1	0.75
	BacT/Alert® *i*AST	10	10	8	6	*5*	1	0.50
	BacT/Alert® *i*LYM	10	8	7	7	*5*	1	0.50
All microorganisms	SCDM	50	48	41	32	*25*	11	–
	BacT/Alert® FA	50	48	40	*31*	20	7	0.3691
	BacT/Alert® SA	50	49	36	*31*	20	9	0.3272
	BacT/Alert® *i*AST	50	50	37	*31*	23	10	0.6241
	BacT/Alert® *i*LYM	50	18	15	13	10	2	< 0.05

Numbers in italics correspond to the LOD value

**Table 3:** Detection frequency of aerobic and anaerobic bacterial growth at five different contamination levels for each culture medium evaluated

Microorganism	Culture medium	*N* per contamination level	Contamination level (CFU/5 mL)					LOD observed (CFU/5 mL)
			2.00	1.00	0.75	0.50	0.25	
*Staphylococcus aureus*	FTM	10	10	7	6	*6*	3	0.50
	BacT/Alert® FN	10	9	7	7	*5*	2	0.50
	BacT/Alert® SN	10	9	5	*5*	4	1	0.75
	BacT/Alert® *i*NST	10	10	6	6	*5*	2	0.50
*Pseudomonas aeruginosa*	FTM	10	7	5	*5*	2	0	0.75
	BacT/Alert® FN	10	7	5	*5*	2	0	0.75
	BacT/Alert® SN	10	7	5	*5*	2	0	0.75
	BacT/Alert® *i*NST	10	7	*5*	4	1	0	1.00
*Bacillus subtilis*	FTM	10	10	7	*6*	3	2	0.75
	BacT/Alert® FN	10	10	8	*6*	3	2	0.75
	BacT/Alert® SN	10	10	7	*5*	3	1	0.75
	BacT/Alert® *i*NST	10	10	8	*7*	4	2	0.75
*Clostridium sporogenes*	FTM	10	10	8	7	*5*	1	0.50
	BacT/Alert® FN	10	10	7	6	*5*	2	0.50
	BacT/Alert® SN	10	9	7	*5*	4	1	0.75
	BacT/Alert® *i*NST	10	10	7	6	*5*	2	0.50
All microorganisms	FTM	40	37	27	*24*	16	6	–
	BacT/Alert® FN	40	36	27	*24*	15	6	0.8652
	BacT/Alert® SN	40	35	24	*20*	13	3	0.1966
	BacT/Alert® *i*NST	40	37	26	*23*	15	6	0.7988

Numbers in italics correspond to the LOD value

Khuu et al. compared the BacT/Alert® system with the official method of the United States Pharmacopeia for sterility testing and observed that the automated system had significantly better performance in microbial detection than the official method [14]. Parveen et al. evaluated the sterility of vaccines and other biological products and found decreased sensitivity of the BacT/Alert® system compared with the traditional method as the microbial load of the samples decreased [1]. However, in the present study, we observed no significant difference (*p* > 0.05) in the performance of the culture media (FTM, BacT/Alert® FN, BacT/Alert® SN, and BacT/Alert® *i*NST) for the detection of *C. sporogenes* and aerobic bacteria nor among the media for detecting aerobic microorganisms (SCDM, BacT/Alert® FA, BacT/Alert® SA, and BacT/Alert® *i*AST).

The BacT/Alert® system was first used in the clinical field for the detection of bacteremia and fungemia, with the introduction of BacT/Alert® SA and BacT/Alert® SN (BacT/Alert® standard media), which use TSB as the base of their formulation. Subsequently, BacT/Alert® FA and BacT/Alert® FN (BacT/Alert® FAN media) supplemented with BHI broth and containing activated carbon were introduced to improve the recovery of fastidious microorganisms from the blood and allow the evaluation of patients during antimicrobial therapy. Several studies have shown significant differences in the ability to detect clinically relevant microorganisms between BacT/Alert® FAN and the BacT/Alert® standard media [15–20].

Weinstein et al. observed that the BacT/Alert® FAN media allowed greater detection of *S. aureus*, coagulase-negative staphylococci, and yeasts (*C. albicans*, *C. parapsilosis*, *T. glabrata*, *Candida tropicalis*, *Candida kruseii*, *Candida guillermondii*, and *Cryptococcus neoformans*) than the BacT/Alert® standard media [19]. Wilson et al. [20] and Mirrett et al. [18] reported that the BacT/Alert® FAN media allowed the detection of a higher number of *S. aureus* isolates and coagulase-negative staphylococci. However, the BacT/Alert® standard media showed better results in the detection of gram-negative non-fermenting bacilli and yeasts (*Candida albicans*, *Candida tropicalis*, and *Candida kruseii*) [18, 20]. Similar results were also reported by Cornish et al. [16]. Bourbeau et al. [15] and Gibb et al. [17] found that the BacT/Alert® FAN media allowed for the detection of more *S. aureus* isolates, whereas Simor et al. [21] and Ericson et al. [22] found that the BacT/Alert® FA media allowed a better ability to detect *Candida* sp. than the BacT/Alert® FN media.

In the present study, no significant difference in detection ability of BacT/Alert® FAN media and BacT/Alert® standard media was observed, although the BacT/Alert® FAN media had a higher frequency of positive results (5.8%) when compared to the BacT/Alert® standard media. Considering each microorganism individually, the BacT/Alert® FAN media allowed for the greater detection of *S. aureus*, *B. subtilis*, and *C. sporogenes* than the BacT/Alert® standard media, whereas the BacT/Alert® standard media allowed greater detection of *P. aeruginosa* and *C. albicans*, but none of these differences were significant (*p* > 0.05).

The BacT/Alert® FAN and BacT/Alert® standard media are directed to the clinical field; the BacT/Alert® *i*AST and *i*NST media were developed to assess the presence of microbial contamination in processed or aseptically prepared food products. Considering the fact that BacT/Alert® *i*AST and *i*NST media have the same base formulations as the standard media, TSB, no significant differences were expected between them. Although, we did not find significant differences in the performance of the BacT/Alert® media compared with the pharmacopoeial media in terms of sterility testing. We found that the BacT/Alert® media allowed for the detection of microbial growth more rapidly than the pharmacopoeial media, SCDM and FTM (Table 4).

**Table 4:** Mean detection time (h) of microbial growth at each contamination level for each culture medium evaluated

Microorganism	Culture medium	Mean detection time (h) at each contamination level (CFU/5 mL)					Mean (h)	Interval (h)
		2.00	1.00	0.75	0.50	0.25		
*Staphylococcus aureus*	SCDM	23.60	23.80	23.71	23.67	23.50	23.66	22.00–24.00
	BacT/Alert® FA	18.18	18.31	18.44	18.91	18.97	18.56	17.66–19.07
	BacT/Alert® AS	18.21	18.39	18.49	18.91	18.94	18.59	17.96–18.99
	BacT/Alert® *i*AST	18.06	18.11	18.23	18.59	18.90	18.38	17.81–19.01
*Pseudomonas aeruginosa*	SCDM	23.11	23.75	23.33	23.60	24.00	23.56	22.00–24.00
	BacT/Alert® FA	18.44	18.52	18.68	18.97	–	18.65	18.32–18.98
	BacT/Alert® AS	18.41	18.47	18.54	19.02	19.87	18.86	18.31–19.87
	BacT/Alert® *i*AST	18.31	18.46	18.88	19.63	20.05	19.07	18.28–20.05
*Bacillus subtilis*	SCDM	24.00	24.00	24.00	24.00	24.00	24.00	24.00–24.00
	BacT/Alert® FA	15.80	16.52	16.69	16.94	17.11	16.61	15.66–17.21
	BacT/Alert® AS	15.81	16.53	16.73	16.98	17.28	16.67	15.68–17.29
	BacT/Alert® *i*AST	15.90	16.80	16.84	16.98	17.23	16.75	15.88–17.25
*Candida albicans*	SCDM	43.00	43.00	43.00	43.00	43.00	43.00	43.00–43.00
	BacT/Alert® FA	30.28	31.53	32.07	32.46	33.26	31.92	30.13–33.26
	BacT/Alert® AS	30.00	30.21	30.78	31.05	31.52	30.71	29.59–31.59
	BacT/Alert® *i*AST	28.46	28.84	29.29	29.43	29.90	29.18	28.00–30.06
	BacT/Alert® *i*LYM	24.48	24.51	25.09	25.91	26.63	25.32	24.00–26.63
*Aspergillus brasiliensis*	SCDM	96.00	96.00	96.00	96.00	96.00	96.00	96.00–96.00
	BacT/Alert® FA	77.02	78.66	79.95	81.03	82.99	79.93	76.82–82.99
	BacT/Alert® AS	77.22	79.28	80.21	82.07	83.05	80.37	76.93–83.05
	BacT/Alert® *i*AST	74.22	74.96	76.44	77.88	79.05	76.51	73.97–79.05
	BacT/Alert® *i*LYM	61.77	61.84	62.15	63.13	64.18	62.61	61.14–64.18
*Staphylococcus aureus*	FTM	23.40	24.00	24.00	23.67	24.00	23.81	22.00–24.00
	BacT/Alert® FN	18.46	18.61	18.84	19.01	19.25	18.83	18.00–19.53
	BacT/Alert® SN	18.55	18.67	18.93	19.22	19.69	19.01	18.48–19.69
	BacT/Alert® *i*NST	18.52	18.57	18.92	19.18	19.54	18.95	18.37–19.59
*Pseudomonas aeruginosa*	FTM	23.14	24.00	24.00	24.00	–	23.79	22.00–24.00
	BacT/Alert® FN	18.60	18.70	19.00	19.20	–	18.87	18.57–19.30
	BacT/Alert® SN	18.59	18.64	18.97	19.26	–	18.86	18.56–19.30
	BacT/Alert® *i*NST	18.31	18.71	19.05	19.05	19.57	18.91	18.28–19.57
*Bacillus subtilis*	FTM	24.00	24.00	24.00	24.00	24.00	24.00	24.00–24.00
	BacT/Alert® FN	21.59	22.99	23.60	23.82	23.99	23.20	21.55–24.00
	BacT/Alert® SN	21.60	23.52	23.82	23.97	24.00	23.38	21.58–24.01
	BacT/Alert® *i*NST	20.22	20.38	20.86	21.77	22.09	21.06	20.19–22.09
*Clostridium sporogenes*	FTM	24.00	24.00	24.00	24.00	24.00	24.00	24.00–24.00
	BacT/Alert® FN	20.70	20.78	20.85	20.96	21.12	20.88	20.68–21.15
	BacT/Alert® SN	20.73	20.85	20.96	21.02	21.29	20.97	20.67–21.29
	BacT/Alert® *i*NST	20.45	20.57	20.66	20.76	21.18	20.72	20.43–21.38

– no growth

Khuu et al. [14], Kielpinski et al. [6], and Parveen et al. [1] performed validation studies of the BacT/Alert® system for sterility of biological products, and Lira et al. [23] studied the implementation of the BacT/Alert® system for sterility testing of injectable products, also observed that the BacT/Alert® system allowed microbial detection after significantly shorter periods than the pharmacopoeial methods for the same microorganisms evaluated in the present study (*S. aureus*, *P. aeruginosa*, *B. subtilis*, *C. sporogenes*, *C. albicans*, and *A. brasiliensis*). Even in the clinical field, the BacT/Alert® system allowed faster detection than the conventional methods, as observed by Alpern et al. [24] and McGowan et al. [25] in studies on the time to positivity of cultures isolated from biological samples (blood) in a pediatric hospital. Simor et al. [21] found a similar result when comparing the BacT/Alert® system with the conventional method for the isolation of pathogens from body fluids of patients undergoing peritoneal dialysis.

In the present study, no significant differences in the detection time of microorganisms (*p* > 0.05) among the BacT/Alert® media was observed, except for BacT/Alert® *i*LYM, which enabled faster detection of *C. albicans* and *A. brasiliensis* than BacT/Alert® SA, FA, and *i*AST (*p* < 0.05).

### Sterility Test

**Fig. 1:**
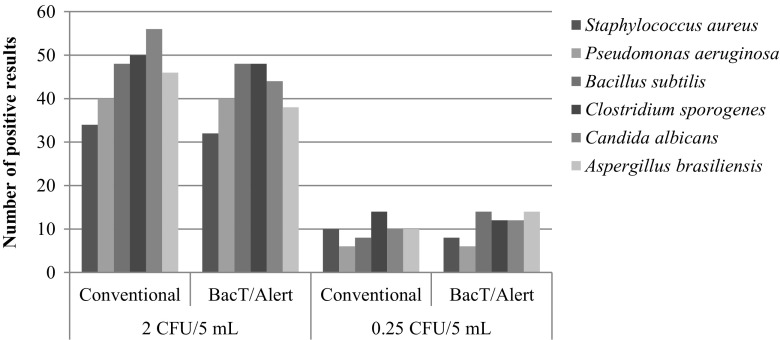
Distribution of the positive results for each level of contamination according to the methodology employed

The number of positive cultures detected with the BacT/Alert® 3D system was compared to the number detected using the compendia method in each microbial load. Figure 1 shows that the absolute number of contaminated samples detected using these methods decreased as the microbial load decreased. The difference between the abilities of these methods to detect microbial contamination was not significant (*p* > 0.05), thus indicating that these two methods have equivalent sensitivities, and these results showed similar with those obtained in other studies that compare the performance of the Bact/Alert® 3D system [1, 12, 14, 26].

**Fig. 2:**
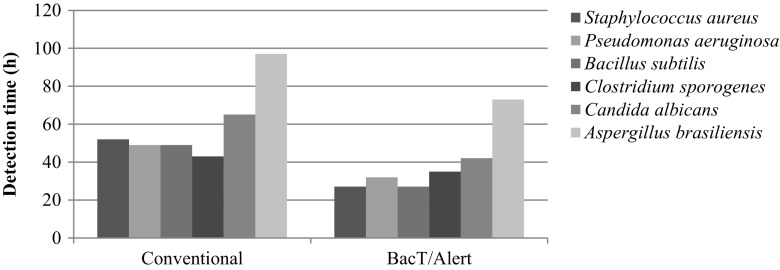
Mean time required to detect microbial growth for each of the methodology employed

Figure 2 presents the mean time required to detect microbial growth, wherein for the Bact/Alert® 3D system the time required to detect the growth included the 18-h incubation before an aliquot was transferred to its culture medium. The data obtained for the 0.2 CFU/5 mL did not evaluate the time of detection, because approximately 82.78% of the assays had no microbial growth at this level of contamination.

Although there are no significant differences between the abilities of the methods to detect bacterial contamination, the BacT/Alert® 3D system detected microbial growth more rapidly than pharmacopoeial method, which indicates superior performance, especially considering that the BacT/Alert® 3D system used an incubation temperature of 32.5 °C to detect all microorganisms evaluated, whereas the conventional method required two incubation temperatures.

## Conclusion

The BacT/Alert® media showed equivalent performance and better mean detection time of microbial growth than the pharmacopoeial media for sterility testing. Although the performance of the BacT/Alert® FAN and standard media did not differ from the BacT/Alert® *i*AST and *i*NST media, the media developed for food products appear to be more suitable for sterility testing, as the BacT/Alert® *i*AST medium features a removable seal that allows the inoculation of solid samples, as well as filter membranes for when membrane filtration sterility testing is necessary.

## References

[1] Parveen S, Kaur S, David SA, Kenney JL, McCormick WM, Gupta RK (2011). Evaluation of growth based rapid microbiological methods for sterility testing of vaccines and other biological products. Vaccine.

[2] Sandle T. Sterility test failure investigations. J GXP Compliance 2012;16(1):1–10.

[3] USP. Validation of alternative microbiological methods. In: United States Pharmacopeia. 40th ed. Rockville: The United States Pharmacopeial Convention, Inc; 2017.

[4] Cundel A. Opportunities for rapid microbial methods. American Pharmaceutical Review. December 2006: http://www.americanpharmaceuticalreview.com/FeaturedArticles/113427-Opportunities-for-Rapid-Microbial-Methods/.

[5] Gray JC, Stärk A, Berchtold M, Mercier M, Neuhaus G, Wirth A. Introduction of a rapid microbiological method as an alternative to the pharmacopoeial method for the sterility test. Am Pharmaceut Rev. 2010;13(6):88–94.

[6] Kielpinski G, Prinzi S, Duguid J, du Moulin G (2005). Roadmap to approval: use of an automated sterility test method as a lot release test for Carticel, autologous cultured chondrocytes. Cytotherapy.

[7] Meder H, Baumstummler A, Chollet R, Barrier S, Kukuczka M, Olivieri F et al. Fluorescence-based rapid detection of microbiological contaminants in water samples. Sci World J 2012;2012:10. doi:10.1100/2012/234858, 1, 10. 10.1100/2012/234858 PMC3353274 22623887

[8] Moldenhauer J, Sutton SV (2004). Towards an improved sterility test. PDA J Pharm Sci Technol.

[9] Miller MJ. Case study of a new growth-based rapid microbiological method (RMM) that detects the presence of specific organisms and provides an estimation of viable cell count. Am Pharmaceut Rev. 2012;15(2):18–25.

[10] Peris-Vicente J, Carda-Broch S, Esteve-Romero J (2015). Validation of rapid microbiological methods. J Lab Autom.

[11] Parenteral Drug Association (PDA). Evaluation, validation and implementation of alternative and rapid microbiological testing methods. Technical Report No. 33 (Revised 2013). Bethesda: Parenteral Drug Association Inc; 2013. p. 59.

[12] Thorpe TC, Wilson ML, Turner JE, DiGuiseppi JL, Willert M, Mirrett S (1990). BacT/Alert: an automated colorimetric microbial detection system. J Clin Microbiol.

[13] Bugno A, Pinto Tde J (2002). Comparative study between culture media employed in sterility test. Boll Chim Farm.

[14] Khuu HM, Stock F, McGann M, Carter CS, Atkins JW, Murray PR, Read EJ (2004). Comparison of automated culture systems with a CFR/USP-compliant method for sterility testing of cell therapy products. Cytotherapy.

[15] Bourbeau P, Riley J, Heiter BJ, Master R, Young C, Pierson C (1998). Use of the BacT/Alert blood culture system for culture of sterile body fluids other than blood. J Clin Microbiol.

[16] Cornish N, Kirkley BA, Easley KA, Washington JA (1999). Reassessment of the routine anaerobic culture and incubation time in the BacT/Alert FAN blood culture bottles. Diagn Microbiol Infect Dis.

[17] Gibb AP, Hill B, Chorel B (1998). Comparative study of BacT/Alert FAN bottles and standard BacT/Alert bottles. Diagn Microbiol Infect Dis.

[18] Mirrett S, Petti CA, Woods CW, Magadia R, Weinstein MP, Reller LB (2004). Controlled clinical comparison of the BacT/ALERT FN and the standard anaerobic SN blood culture medium. J Clin Microbiol.

[19] Weinstein MP, Mirrett S, Reimer LG, Wilson ML, Smith-Elekes S, Chuard CR, Joho KL, Reller LB (1995). Controlled evaluation of BacT/Alert standard aerobic and FAN aerobic blood culture bottles for detection of bacteremia and fungemia. J Clin Microbiol.

[20] Wilson ML, Weinstein MP, Mirrett S, Reimer LG, Feldman RJ, Chuard CR, Reller LB (1995). Controlled evaluation of BacT/alert standard anaerobic and FAN anaerobic blood culture bottles for the detection of bacteremia and fungemia. J Clin Microbiol.

[21] Simor AE, Scythes K, Meaney H, Louie M (2000). Evaluation of the BacT/Alert microbial detection system with FAN aerobic and FAN anaerobic bottles for culturing normally sterile body fluids other than blood. Diagn Microbiol Infect Dis.

[22] Ericson EL, Klingspor L, Ullberg M, Ozenci V (2012). Clinical comparison of the Bactec Mycosis IC/F, BacT/Alert FA, and BacT/Alert FN blood culture vials for the detection of candidemia. Diagn Microbiol Infect Dis.

[23] Lira RS, Bugno A, Oliveira WA, Almodovar AA, Saes DP, Pinto Tde J (2015). Application of the BacT/ALERTR 3D system for sterility testing of injectable products. Braz J Microbiol.

[24] Alpern ER, Alessandrini EA, Bell LM, Shaw KN, McGowan KL (2000). Occult bacteremia from a pediatric emergency department: current prevalence, time to detection, and outcome. Pediatrics.

[25] McGowan KL, Foster JA, Coffin SE (2000). Outpatient pediatric blood cultures: time to positivity. Pediatrics.

[26] Yonetani S, Okazaki M, Araki K, Makino H, Fukugawa Y, Okuyama T, Ohnishi H, Watanabe T (2012). Direct inoculation method using BacT/ALERT 3D and BD Phoenix System allows rapid and accurate identification and susceptibility testing for both Gram-positive cocci and Gram-negative rods in aerobic blood cultures. Diagn Microbiol Infect Disease.

